# Immune Response and Risk of Decompensation following SARS-CoV-2 Infection in Outpatients with Advanced Chronic Liver Disease

**DOI:** 10.3390/ijms25158302

**Published:** 2024-07-30

**Authors:** Anna Brujats, Anna Huerta, Rubén Osuna-Gómez, Albert Guinart-Cuadra, Andreu Ferrero-Gregori, Clàudia Pujol, German Soriano, Maria Poca, Javier Fajardo, Angels Escorsell, Adolfo Gallego, Silvia Vidal, Càndid Villanueva, Edilmar Alvarado-Tapias

**Affiliations:** 1 Department of Gastroenterology and Hepatology, Hospital de la Santa Creu i Sant Pau, Biomedical Research Insitute Sant Pau (IIB Sant Pau), 08041 Barcelona, Spain; abrujats@santpau.cat (A.B.); ahuerta@santpau.cat (A.H.); andreu.ferrero.gregori@gmail.com (A.F.-G.); cpujol@santpau.cat (C.P.); gsoriano@santpau.cat (G.S.); mpoca@santpau.cat (M.P.); jfajardo@santpau.cat (J.F.); mescorsell@santpau.cat (A.E.); agallego@santpau.cat (A.G.); cvillanueva@santpau.cat (C.V.); 2 Departament Medicina UAB, Universitat Autònoma de Barcelona, 08193 Barcelona, Spain; 3 Inflammatory Diseases Department, Institut Recerca Hospital de la Santa Creu i Sant Pau (IR Sant Pau), 08041 Barcelona, Spain; aguinart@santpau.cat (A.G.-C.); svidal@santpau.cat (S.V.); 4 Centre for Biomedical Research in Liver and Digestive Diseases Network (CIBERehd), Instituto de Salud Carlos III, 28029 Madrid, Spain

**Keywords:** advanced chronic liver disease (ACLD), decompensation, SARS-CoV-2 infection, immune response, anti-Spike 1 immunoglobulin G

## Abstract

Advanced chronic liver disease (ACLD) is associated with a wide spectrum of immune dysfunction. The clinical impact of SARS-CoV-2 on the development of decompensation and immune response in unvaccinated outpatients has not as yet been clearly defined. This study aimed to evaluate the clinical and immunological impact of SARS-CoV-2 on outpatients with ACLD. This is an observational case–control study, in which ACLD outpatients were included prospectively and consecutively and classified into two groups: SARS-CoV-2 infected and non-infected. Patients’ baseline characteristics and infection data were collected and analyzed. Immunoglobulin G (IgG) levels against Spike 1 were evaluated. The primary endpoint was risk of liver decompensation during follow-up, assessed after propensity score matching and adjusted by Cox regression. Between October 2020 and July 2021, ACLD outpatients (n = 580) were identified, and 174 patients with clinical follow-up were included. SARS-CoV-2 infection incidence was 7.6% (n = 44). Risk of liver decompensation was significantly higher after infection (HR = 2.43 [1.01–5.86], *p* = 0.048) vs. non-infection. The time of IgG evaluation was similar in all patients (n = 74); IgG concentrations were significantly higher in compensated vs. decompensated patients (1.02 ± 0.35 pg/mL vs. 0.34 ± 0.16 pg/mL, *p* < 0.0001) and correlated with hemoglobin levels. The dysregulation of the innate immune response in patients with decompensated liver disease increased the risk of further decompensation following SARS-CoV-2, mainly due to a worsening of ascites.

## 1. Introduction

Advanced chronic liver disease (ACLD) is associated with an increased risk of liver decompensation, including the development of infections, which is associated with higher morbidity and mortality. Cirrhosis-associated immune dysfunction (CAID) is associated with a wide spectrum of immune modifications. CAID includes two main changes: immunodeficiency due to an impaired response to pathogens and systemic inflammation as a consequence of persistent and inadequate stimulation of the systemic cells. The intensity of these two conditions varies depending on the stage of cirrhosis and the presence of incidental events, such as bacterial infections [1,2].

CAID can be characterized in terms of two different immune phenotypes: The low-grade systemic inflammatory phenotype is present in patients with compensated liver disease and in those with decompensated liver disease without organ failure. This phenotype includes an increased expression of surface activation antigens in circulating immune cells and the production of pro-inflammatory cytokines [3,4,5,6]. When cirrhosis progresses to a decompensated state, CAID is more pronounced and the immune response to the persistent bacterial challenge becomes increasingly impaired [7,8]. The second phenotype is a high-grade systemic inflammatory phenotype that eventually appears in patients with acute-on-chronic liver failure (ACLF) and is characterized by an excessive compensatory anti-inflammatory response and the dysregulation of immune effector cells [9,10,11,12].

In advanced stages of liver disease, bacterial infections are one of the main causes of liver decompensation [1,13]. However, the impact of viral infections such as SARS-CoV-2 infection has been little studied in the overall ACLD population [14]. A multicentric study focused on patients with non-alcoholic fatty liver disease (NAFLD) cirrhosis reported similar liver-related outcomes before the pandemic and in the following year [15]. Nevertheless, the impact of SARS-CoV-2 infection on the immunity and clinical decompensations of unvaccinated ACLD outpatients is currently unclear.

There are two phases in a SARS-CoV-2 infection [16]. The first, which is clinically mild, involves the host’s innate immune response, while the second involves a specific adaptive response. However, if the immune response is impaired, in the second phase, which is clinically severe, the virus spreads, causing a massive destruction of affected tissues, especially in organs with a high expression of angiotensin-converting enzyme 2 (ACE2). The affected cells induce hyperinflammation, triggering dysfunction in multiple organs, especially in the lung and the liver [16,17,18,19]. In patients without underlying liver disease, liver damage is usually mild and reversible, but in patients with ACLD, the infection can lead to liver dysfunction, mediated by the immune response, microvascular thrombosis, and alterations to the gut–liver–brain axis, among others [20,21,22,23]. It has been suggested that liver damage in patients with ACLD may be more pronounced due to CAID [24].

On the other hand, antibody production by the host after exposure to SARS-CoV-2 largely depends on the integrity of the immune system. Immunoglobulin M is the first antibody to appear in response to the initial exposure to antigens from SARS-CoV-2 proteins (against Spike 1), and plays a critical role in the primary immune response [16]. This response can be impaired in patients with ACLD [25].

Mortality from SARS-CoV-2 infection in patients with liver disease has been evaluated in several studies [21,24,26,27,28], most of which have evaluated the impact of SARS-CoV-2 infection in hospitalized patients. However, there are insufficient data regarding the impact of SARS-CoV-2 infection on further decompensating events in unvaccinated outpatients with ACLD and its relationship with the innate immune response.

This study focuses on characterizing, from an epidemiological, clinical, and immunological perspective, the effects of SARS-CoV-2 infection on unvaccinated ACLD outpatients and their decompensation events compared with patients without a SARS-CoV-2 infection.

## 2. Results

### 2.1. Study Population

**Figure 1:**
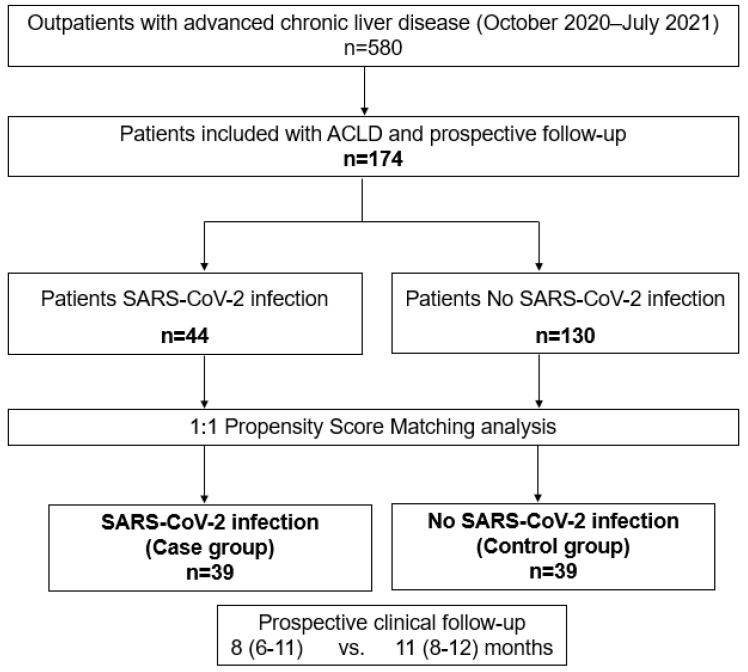
Study flowchart: A total of 580 outpatients with advanced chronic liver disease (ACLD) were evaluated from October 2020 to July 2021. Out of them, 174 patients (30%) met the study inclusion criteria and were included. After a 1:1 propensity score matching analysis, two groups of study were identified.

A total of 580 outpatients with ACLD were evaluated from October 2020 to July 2021, of whom 174 patients (30%) met the study inclusion criteria and were included in this study after informed consent was signed and were prospectively followed-up. Immunoglobulin G (IgG) levels were positive and quantified in 44 patients with SARS-CoV-2 infection, representing an incidence rate of 7.6% out of the total outpatient population. A study flowchart is depicted in Figure 1.

### 2.2. Characterization of Infected and Non-Infected Patients

Two groups of patients were identified after the clinical and serological analysis: patients with SARS-CoV-2 infection (infected group, n = 44) and patients without SARS-CoV-2 infection (non-infected group, n = 130).

Within the infected group (n = 44) (primary SARS-CoV-2 infection without vaccination), most patients were male (61%), had alcohol-associated liver disease (ALD) (36%), and had active alcohol intake (34%). Cardiovascular risk factors such as diabetes mellitus (41%) and arterial hypertension (48%) were slightly more prevalent in the infected group, although only obesity (43% vs. 23%, *p* = 0.010) was significantly higher in the infected group. Liver function characterized by the Child–Pugh score (6.1 ± 1.2 vs. 5.7 ± 1.1, *p* = 0.06) and MELD score (8.8 ± 3.1 vs. 8.6 ± 2.6, *p* = 0.804), analytical parameters, and liver elastography by FibroScan were similar in both groups. Hemoglobin was significantly lower in infected patients, however without clinically relevant anemia. In the infected group, 18 patients had compensated ACLD and 26 had decompensated ACLD. After the complete prospective follow-up (FU), no differences were observed in the number of months of FU between the two groups (8 [6,7,8,9,10,11] vs. 11 [8,9,10,11,12] months). At the end of the FU, the incidence of decompensation was 16 patients (36%) in the infected group vs. 10 (8%) in the non-infected group (*p* < 0.001). There was a significantly higher mortality rate in the infected group vs. the non-infected group (14 patients [32%] vs. 5 [4%], *p* < 0.001). The most common causes of death in the infected group were as follows: six were liver-related (43%), three were hepatocarcinoma (HCC) (21%), three were non-liver-related (21%), and two were SARS-CoV-2 infection (14%); in the non-infected group, three were liver-related (60%), one was non-liver-related (20%), and one was cholangiocarcinoma (20%). These data are shown in Table 1.

In the infected group, the severity of infection symptoms was different between compensated and decompensated patients, with the compensated patients experiencing slightly more severe symptoms than the decompensated patients (44% vs. 27%, *p* = 0.021): fever (50% vs. 34%), cough, and shortness of breath (39% vs. 27%) were the most frequent symptoms.

Hospital admission during infection was required in 21 patients (47.7%); three (7%) of whom required intensive care unit (ICU) admission, only one patient (2%) required orotracheal intubation (OTI), and two patients died during the infection (4.5%). No differences were observed in the proportion of hospital admissions (56% vs. 42%, *p* = 0.387), ICU admission (6% vs. 8%, *p* = 0.820), OTI (0% vs. 4%), or death (0% vs. 4%, *p* = 1.00) between compensated and decompensated patients. In the 1-year FU, a total of 16 patients (36%) had liver-related clinical decompensation (mainly due to ascites) in the infected group vs. 10 patients (8%) in the non-infected patients (*p* < 0.001) (OR, 6.86 [95% CI: 2.81–16.71]). No differences were observed in the incidence of liver-related decompensation between patients with compensated and previous decompensated ACLD (12% vs. 26%, *p* = 0.233).

**Figure 2:**
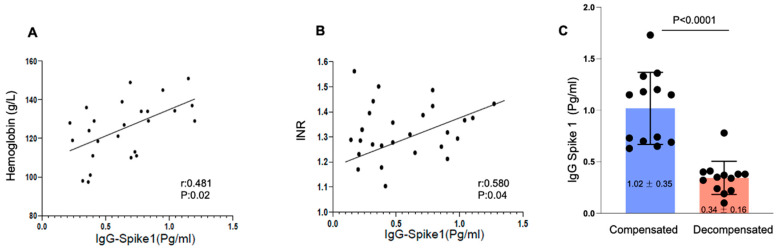
Biological relation between immunoglobulin G (IgG) levels against Spike 1 (IgG Spike 1) and analytical and clinical liver stage: Results are expressed as Spearman correlations and *p*-values and mean ± standard deviation in the IgG concentrations. (**A**) Correlation between hemoglobin levels and IgG Spike 1 in overall patients. (**B**) Correlation between International Normalized Ratio (INR) levels and IgG Spike 1 in overall patients. (**C**) IgG levels against Spike 1 of SARS-CoV-2 in patients with SARS-CoV-2 infection according to a previous decompensation of advanced chronic liver disease (ACLD).

The moment of IgG anti-Spike 1 determination between compensated and decompensated patients considering the date of the SARS-CoV-2 infection was similar in both groups (day 35, IQR 26.5–47 vs. day 42, IQR 30.75–46; *p* = 0.575). IgG anti-Spike 1 concentrations (pg/mL) in plasma were only evaluated in 26 patients, and the IgG values were positively correlated with the hemoglobin (g/L) (r: 0.48, *p* = 0.02) and International Normalized Ratio (INR) (r: 0.58, *p* = 0.04). No correlation was observed with the concentrations of innate immune cells (absolute concentrations of lymphocytes, monocytes, neutrophils, and platelets) or with liver function scores (Child–Pugh, MELD). IgG anti-Spike 1 was significantly higher in compensated patients than in decompensated patients (1.02 ± 0.35 pg/mL vs. 0.34 ± 0.16 pg/mL, *p* < 0.0001). These data are shown in Figure 2A–C.

### 2.3. Clinical Characteristics and Outcomes in Matched Populations

**Figure 3:**
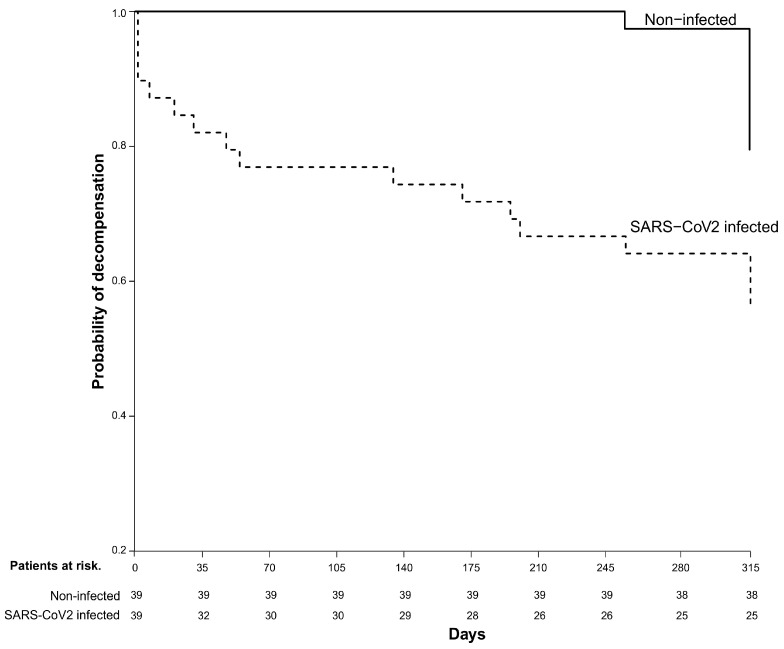
Probability of liver decompensation in patients with ACLD–non-infected vs. SARS-CoV-2-infected patients in matched data. After matching, the probability of liver-related decompensation was compared between SARS-CoV-2-infected and non-infected patients, using a stratified Cox regression model, accounting for matched pairs as a stratum. The Cox regression results are reported as a hazard ratio (HR) with 95% confidence intervals.

After PS matching, the final study cohort included 39 cases in the SARS-CoV-2-infected group and 39 controls in the non-infected group. The two groups had similar baseline demographic and clinical characteristics and were comparable in terms of age, sex, liver function, liver elastography by FibroScan, and the proportion of patients with previous liver-related decompensation. These data are shown in Table 2. The risk of developing liver-related decompensation during the 1-year FU period in the final matched cohort was significantly higher in the infected group in than in the non-infected group (44% vs. 21%, *p* = 0.03 by the log-rank test), with ascites being the most frequent liver-related decompensation (56%). In the Cox regression analysis, risk of decompensation was twofold in the infected group (HR = 2.43 (95% CI 1.01–5.86, *p* = 0.048)), mainly during the first 60 days after the infection. These data are shown in Figure 3. The diagnostic performance of SARS-CoV-2 infection for the prediction of decompensation was evaluated using a receiver operating characteristic (ROC) curve analysis. The discriminative ability of the SARS-CoV-2 infection was a moderately good predictor of decompensation (AUC 0.72; 95% CI 0.62–0.82), with a sensitivity of 0.615 (0.406, 0.798) and a specificity of 0.822 (0.750, 0.880), to identify patients with decompensation during FU (Supplementary Figure S1).

## 3. Discussion

This study showed that SARS-CoV-2 infection in non-vaccinated ACLD outpatients predisposes them to liver-related decompensation, particularly ascites, mainly within the first 60 days after infection. The levels of IgG against Spike 1 were related to the liver disease stage (compensated or decompensated) and correlated with hemoglobin levels and INR.

Previous series have assessed the impact of SARS-CoV-2 infection on patients with ACLD in Eastern populations, with demographic differences and in different etiologies of liver disease [15,19,21]. However, there are few studies evaluating the impact of the infection on unvaccinated ambulatory patients with ACLD during the first year of the pandemic. While the true global incidence of SARS-CoV-2 infection in ACLD outpatients’ specific population remains poorly defined, in our study, the incidence of SARS-CoV-2 infection in ACLD outpatients at a tertiary hospital was 7.6%. SARS-CoV-2 infection was independent of the clinical staging of ACLD, although it is important to note that the included patients were ambulatory patients, though the majority had a Child–Pugh score of A or B and a MELD score < 10. Most SARS-CoV-2-infected patients were male, with ALD, metabolic syndrome, and obesity. These factors were accentuated during the period of pandemic restrictions due to an increase in alcohol consumption and a reduction in mobility and physical activities in the population. In this study, these two factors were associated with worse clinical outcomes. However, in our study, the proportion of patients requiring hospital admission, ICU admission, and OTI was similar in compensated and decompensated patients, in agreement with previous studies [29].

In this study, we identified a positive correlation between IgG anti-Spike 1 concentration and hemoglobin levels in both compensated and decompensated ACLD patients. We could hypothesize that, in ACLD patients, independent of the disease stage, as has been suggested in the general population, there is an interference of specific viral proteins with the 1-β chain of hemoglobin, which could impair heme metabolism and oxygen transport. Previous studies have raised the possibility that SARS-CoV-2 attacks the 1-beta globin chain, capturing porphyrin by means of structural and non-structural proteins to dissociate it from iron. This metabolic alteration may lead to an accumulation of free iron, resulting in an elevated C-reactive protein (CRP), an inflammatory marker, leaving porphyrin available to the virus [30]. Several theories have been proposed as possible mechanisms by which SARS-CoV-2 could assault the hemoglobin chain: through protein S to ACE2 receptors expressed on plasma cells; through an interaction between its S-CD147 protein and the erythrocyte membrane; and through the action of the proteins of the virus, such as nsp16-nsp10, ORF3, and ORF10, which interact with beta chain hemoglobin to dissociate iron and form porphyrin, resulting in hemoglobin dysfunction, with lung cells failing to exchange carbon dioxide and oxygen [31].

In our study, we also observed a significant positive correlation between IgG anti-Spike 1 concentration and INR (prothrombin time) independent of the stage of liver disease. It is likely that this finding is related to the impact on the coagulation cascade of the immune response triggered by the viral infection. Several studies found that an inflammatory condition in a SARS-CoV-2 infection causes a disruption to hemostasis and significant alterations in coagulation parameters, which can result in thromboses and is linked to poor outcomes [32]. There is evidence that prothrombin time, one of the most commonly used measurements of coagulation in the clinic, especially in patients with liver disease, is longer in patients with severe illness than in patients with non-severe illness, and it is associated with higher mortality [33].

Regarding the clinical manifestations of SARS-CoV-2 infections, the most frequent symptoms in ACLD ambulatory patients were fever and cough, as in the general population without liver disease [17,34]. Nevertheless, it is important to emphasize that clinical symptoms were less frequent in patients with decompensated cirrhosis, although no statistically significant differences were found. These findings may be related to an initial response to SARS-CoV-2 infection that was less aggressive in decompensated patients due to the impaired innate immune response in the context of their dysregulated immune system [22,23,35,36]. The immune response to SARS-CoV-2 is mediated through the production of IgG antibodies against Spike 1 of the virus by B lymphocytes. The lower levels of IgG against Spike 1 observed in decompensated patients in our study may help to explain the presence of less severe clinical symptoms in this group.

During the 1-year FU of all the included patients, SARS-CoV-2 infection increased the risk of decompensation in both compensated and decompensated patients compared with non-SARS-CoV-2-infected ACLD patients. However, a higher risk of further decompensations was found in decompensated patients than in compensated patients, mainly in the first 60 days following infection. This may be explained by a greater disruption of the baseline innate immune response due to the stage of liver disease, which worsens even more after SARS-CoV-2 viral infection, mainly in patients with decompensated ACLD, potentially leading to a worse prognosis and a higher rate of further decompensation in this subgroup of patients [37,38,39]. The incidence of liver decompensation beyond the first 60 days after the infection was likely more closely related to the progression of ACLD rather than the SARS-CoV-2 infection per se. In fact, in our study, there was a significantly higher mortality in the infected than in the non-infected group due to liver-related causes and HCC. This is in line with previous studies, although most of them included patients who required hospital admission [24].

This study has several limitations. First, it is a single-center study, which involved significant limitations in patient inclusion during the pandemic. During the inclusion and data collection processes, there were two periods with a higher incidence of SARS-CoV-2 infections, restricting accessibility to outpatient visits and possibly leading to the non-inclusion of some patients. Second, vaccination against SARS-CoV-2 was started during the inclusion process. Patients who had already been vaccinated were excluded, potentially resulting in unreported SARS-CoV-2-infected patients. Third, there was also a likely underdiagnosis of infection in asymptomatic patients without positive IgG serologies. Finally, the sample size of SARS-CoV-2-infected patients is small. However, we used a PS analysis that allowed us to balance both groups according to the main different baseline characteristics (SARS-CoV-2-infected group vs. non-infected group). With this type of statistical analysis, we identified that SARS-CoV-2 infection doubles the risk of liver-related decompensation. However, probably because of the small sample size, other evaluated variables, such as symptomatology, did not reach statistical significance.

International guidelines have established and recommended general measures mainly for patients with ACLD or liver transplant recipient patients infected by SARS-CoV-2 [40,41,42]. However, although ACLD patients should be considered a high-priority population for vaccination, international vaccine coverage remains suboptimal [43,44]. Therefore, these results indicate that, in the context of possible new viral infection epidemics, whether by SARS-CoV-2 or not, ACLD patients with better liver function under outpatient FU, but with prior decompensation, may benefit from a better diagnostic strategy, closer monitoring, and more intensive preventive measures to avoid infection and a worse liver disease progression.

In conclusion, patients with ACLD, especially those with prior decompensation ACLD, male gender, ALD etiology, and obesity, had a higher risk of decompensation following SARS-CoV-2 infection, mainly during the first 60 days, with ascites being the most frequent decompensation and possibly related to a low-grade immune response.

## 4. Materials and Methods

### 4.1. Study Design and Participants

This is a case–control, single-center, observational study performed at Santa Creu i Sant Pau Hospital in Barcelona (Spain), a tertiary hospital without liver transplantation facilities. The study protocol, which fulfilled the Guidelines for Good Clinical Practice in observational studies, was conducted in accordance with the Declaration of Helsinki and was approved by the institutional review board (CEIm, Sant Pau) on 11 June 2020 with the approval code 20/186. Written informed consent was obtained from all patients. All the authors vouch for the integrity and accuracy of the analysis and its fidelity to the protocol.

Patients with a diagnosis of ACLD of any etiology, followed-up in the outpatient clinic before the SARS-CoV-2 vaccination period, were eligible for inclusion. The total number of outpatients in our center was determined based on the data of the outpatient programmation department (eight different agendas), in which those patients with a diagnosis of ACLD (compensated and decompensated) were identified.

Among the main inclusion criteria considered were the following: outpatients with ACLD diagnosed by a previous liver biopsy or by compatible clinical, biochemical, and ultrasonography findings, without SARS-CoV-2 vaccination and with at least 90 days of prospective FU. Patients with any of the following criteria were excluded: hospitalized patients, age < 18 or >85 years, Child–Pugh score > 12, previous infection by SARS-CoV-2, previous vaccination for SARS-CoV-2, HCC out of Milan criteria, comorbidities with life expectancy of <12 months, non-cirrhotic portal hypertension, pregnancy, or lactation.

From October 2020 to July 2021, 580 patients with ACLD were identified, from whom only eligible patients were consecutively and prospectively included after informed consent was obtained. Finally, in this study, 174 patients were included with a prospective clinical FU.

### 4.2. Data Collection and Serological Study

Baseline epidemiological, clinical, and analytical data related to ACLD were collected from all included patients (n = 174). Moreover, during the clinical visit, a questionnaire was given, which included all the items regarding the date of SARS-CoV-2 infection diagnosis by real-time reverse transcription–polymerase chain reaction and the signs and symptoms of SARS-CoV-2 infection (Supplementary Table S1). Among them, IgG against a specific protein, Spike 1, was analyzed in blood samples in only 74 patients by the standard laboratory of liver disease FU, in accordance with the hospital’s restrictions during the SARS-CoV-2 pandemic.

After the completed clinical FU and the specific interview regarding SARS-CoV-2 during the outpatients’ visits and serologic results (IgG), two groups were defined: one group of patients with SARS-CoV-2 infection and positive IgG against SARS-CoV-2 (the infected group) and a second group of patients not infected with SARS-CoV-2 (non-infected group).

In the infected group, all the included patients had a very well-characterized SARS-CoV-2 infection, including the date of infection, the symptomatology displayed during the infection, the need for medical treatment or hospital admission, clinical liver decompensation during the infection, and further decompensation during the 12 months of prospective FU.

In both groups, clinical liver-related decompensations were classified according to the BAVENO VII criteria (ascites, hepatic encephalopathy, and variceal bleeding) [45].

The quantification of the IgG levels was performed by a specific ELISA (pg/mL) (SARS-CoV-2 NP IgG ELISA Kit Catalog Number MBS398004), MyBioSource, San Diego, CA, USA, on 74 patients with positive IgG against the Spike 1 protein; however, only the IgG values of the matched cohort were considered for the correlation analysis. All the samples for the IgG quantification were obtained between day 25 and day 45 of the SARS-CoV-2 infection in all the included patients.

### 4.3. Statistical Analysis

Categorical variables were reported as absolute (n) and relative frequencies (%), whereas continuous variables were reported as mean ± standard deviation or median (IQR). For group comparisons of normally distributed variables, the Student’s *t* test was used, while the Mann–Whitney *U* test was used for non-normally distributed variables. To account for the non-random selection of groups, a 1:1 propensity score (PS) was performed with a caliper with ≤0.1 standard deviations of the logit of the PS. The variables included for the match were age, sex, liver disease etiology, previous decompensation, active alcohol consumption, body mass index, diabetes mellitus, arterial hypertension, dyslipidemia, Child–Pugh, and MELD score. The balance of matched variables was determined by using the standardized difference, with values < 10 being the threshold for a quality match.

After matching, the outcomes of interest were compared between SARS-CoV-2 infected and non-infected patients, using a stratified Cox regression model, accounting for matched pairs as a stratum. The Cox regression results are reported as a hazard ratio (HR) with 95% confidence intervals.

ROC curve analysis was used to assess the diagnostic accuracy of SARS-CoV-2 infection to predict decompensation during FU.

Differences were considered statistically significant at the level of <0.05. All statistical analyses were performed using SPSS Statistics 25, R 4.2, and GraphPad Prism10.2.3.

## Figures and Tables

**Table 1:** Baseline characteristics and follow-up in patients infected with SARS-CoV-2 and non-infected with SARS-CoV-2.

	Non-Infected SARS-CoV-2 (n = 130)	Infected SARS-CoV-2 (n = 44)	*p* Value
Sex, M/F, n (%)	93 (71)/37 (29)	27 (61)/17 (39)	0.207
Age, (yr)	65.1 ± 9.9	64.3 ± 10.3	0.648
Liver disease etiology, n (%)			
Alcohol	59 (45)	16 (36)	
Virus (HBV/HCV)	26 (20)	9 (21)	
Alcohol + Virus	12 (9)	8 (18)	0.241
MASLD	15 (12)	2 (4)	
Others	18 (14)	9 (21)	
Previous decompensation, ** n (%)	73 (56)	26 (59)	0.734
Active alcohol intake, n (%)	18 (14)	15 (34)	0.003
Diabetes mellitus, n (%)	46 (35)	18 (41)	0.511
Arterial hypertension, n (%)	51 (39)	21 (48)	0.323
Dyslipidemia, n (%)	29 (22)	14 (32)	0.206
Body mass index (BMI), (kg/m^2^)	27.1 ± 4.3	29 ± 5.6	0.017
Obesity, n (%)	30 (23)	19 (43)	0.010
Active smoking, n (%)	40 (31)	9 (20)	0.189
Child-Pugh score, (points)	5.7 ± 1.1	6.1 ± 1.2	0.060
MELD score, (points)	8.6 ± 2.6	8.8 ± 3.1	0.804
Bilirubin, (µmol/L)	22.9 ± 14.9	26.5 ± 17.5	0.199
Creatinine, (µmol/L)	78.5 ± 19.8	79.8 ± 24.8	0.708
Prothrombin time, (INR)	1.14 ± 0.2	1.22 ± 0.3	0.049
Albumin, (g/L)	38.6 ± 5.2	36.6 ± 6.2	0.029
Platelet count, ×10^−3^	131 ± 74	109 ± 62	0.073
Hemoglobin, (g/L)	135 ± 22	125 ± 21	0.010
Aspartate aminotransferase, (U/L)	36 ± 23	40 ± 22	0.459
Alanine aminotransferase, (U/L)	29 ± 20	39 ± 22	0.357
Gamma glutamyltranspeptidase, (U/L)	157 ± 331	129 ± 142	0.595
Alkaline phosphatase, (U/L)	119 ± 59	122 ± 60	0.783
Liver stiffness, (kPa)	23.9 ± 20.1	20.1 ± 14.6	0.410
Esophageal varices small/large, n (%)	30 (23)/62 (48)	13 (29)/23 (5)	0.706
Death during follow-up (FU), n (%)	5 (4)	14 (32)	0.001
Decompensation during FU/after SARS-CoV-2 infection, n (%)	10 (8)	16 (36)	0.001

Categorical variables (%) were compared using the Fisher exact test. Continuous variables (mean values ± SD), were compared using the unpaired Student’s test or the non-parametric Mann–Whitney rank-sum test. ** Patients with previous clinical ascites, at least one previous episode of encephalopathy ≥ grade II of West Haven, or variceal bleeding. BMI: body mass index; FU: follow-up; HBV: hepatitis B virus; HCV: hepatitis C virus; INR: International normalized ratio; MASLD: metabolic dysfunction-associated steatotic liver disease; MELD: model for end-stage liver disease, kPa: kilopascal.

**Table 2:** Balanced baseline characteristics in patients infected with SARS-CoV-2 and not infected with SARS-CoV-2 after propensity score matching *.

	Non-Infected SARS-CoV-2 (n = 39)	Infected SARS-CoV-2 (n = 39)	*p* Value	Std. Mean Difference
Sex, M/F, n (%)	13 (33)/26 (67)	15 (38)/24 (62)	0.637	0.105
Age, (yr)	64.1 ± 9.2	64.1 ± 10.5	0.968	−0.002
Liver disease etiology, n (%)				
Alcohol	16 (41)	14 (36)		−0.107
Virus (HBV/HCV)	10 (26)	8 (21)		−0.127
Alcohol + Virus	4 (10)	6 (15)	0.887	0.133
MASLD	1 (3)	2 (5)		0.123
Others	8 (21)	9 (23)		0.064
Previous decompensation, ** n (%)	23 (59)	22 (56)	0.819	−0.052
Active alcohol consumption, n (%)	11 (28)	10 (26)	0.799	−0.054
Diabetes mellitus, n (%)	18 (46)	17 (44)	0.820	−0.052
Arterial hypertension, n (%)	19 (49)	17 (44)	0.650	−0.103
Dyslipidemia, n (%)	13 (33)	12 (31)	0.808	−0.055
BMI, (kg/m^2^)	28.1 ± 4.6	28.4 ± 5.5	0.859	−0.065
Child-Pugh score, (points)	6.2 ± 1.3	6.1 ± 1.2	0.872	−0.087
MELD score, (points)	9.6 ± 3.0	8.8 ± 3.2	0.133	−0.255
Bilirubin, (µmol/L)	26.7 ± 17.9	26.4 ± 17.4	0.913	−0.016
Creatinine, (µmol/L)	82.6 ± 27.2	81.4 ± 25.7	0.964	−0.047
Prothrombin time, (INR)	1.21 ± 0.3	1.24 ± 0.4	0.692	0.072
Albumin, (g/L)	38.2 ± 5.2	36.7 ± 6.3	0.237	−0.238
Platelet count, ×10^−3^	143 ± 87	109 ± 64	0.051	−0.559
Hemoglobin, (g/L)	130 ± 20	124 ± 22	0.190	−0.270
Aspartate aminotransferase, (U/L)	41 ± 27	40 ± 22	0.459	−0.043
Alanine aminotransferase, (U/L)	30 ± 18	31 ± 22	0.968	0.042
Gamma glutamyltranspeptidase, (U/L)	246 ± 485	133 ± 145	0.241	−0.815
Alkaline phosphatase, (U/L)	136 ± 57	126 ± 63	0.478	−0.172
Liver stiffness, (kPa)	27.1 ± 18.5	24.9 ± 12.5	0.879	−0.191

Data are presented as proportion (%) and continuous variables (Mean ± Standard Deviation). * Matched data: A 1:1 propensity score was performed. The variables included for the match were: age, sex, liver disease etiology, previous decompensation, active alcohol consumption, body mass index (BMI), diabetes mellitus, arterial hypertension, dyslipidemia, Child-Pugh and MELD score. ** Patients with previous clinical ascites, at least one previous episode of encephalopathy ≥ grade II of West Haven, or variceal bleeding. BMI: body mass index; HBV: hepatitis B virus; HCV: hepatitis C virus; INR: International normalized ratio; MASLD: metabolic dysfunction-associated steatotic liver disease; MELD: model for end-stage liver disease. Std. Mean Difference: standard mean difference, kPa: kilopascal.

## Data Availability

The raw data supporting the conclusions of this article will be made available by the corresponding authors (ealvaradot@santpau.cat, rosuna@santpau.cat) on request.

## Supplementary Materials

The following supporting information can be downloaded at https://www.mdpi.com/article/10.3390/ijms25158302/s1.

## Abbreviations

ACE 2: angiotensin-converting enzyme 2; ACLD: advanced chronic liver disease; ACLF: acute-on-chronic liver failure; ALD: alcohol-associated liver disease; CAID: cirrhosis-associated immune dysfunction; BMI: body mass index; FU: follow-up; HCC: hepatocellular carcinoma; HR: hazard ratio; ICU: intensive care unit; IgG: immunoglobulin G; INR: International Normalized Ratio; MELD: model for end-stage liver disease; MASLD: metabolic dysfunction-associated steatotic liver disease; NAFLD: non-alcoholic fatty liver disease; OTI: orotracheal intubation; PS: propensity score match; ROC: receiver operating characteristic.
