# Supplementary material for: Immune Response and Risk of Decompensation following SARS-CoV-2 Infection in Outpatients with Advanced Chronic Liver Disease
Source: Int J Mol Sci. 2024 Jul 30;25(15):8302. doi: 10.3390/ijms25158302 (PMC11312207; doi:10.3390/ijms25158302)
Supplement: Supplementary file 1 [file ijms-25-08302-s001.zip › ijms-3101037-supplementary.pdf]

Supplementary Material

Table S1. Questionary of symptoms of SARS-CoV-2 infection.

Name:

Date of interview: *Date of infection:*

SANT PAU Campus Salut Barcelona   Hospital de la Santa Creu i Sant Pau

- Did you have a COVID-19 infection?
- When was the infection diagnosed?
- Which was the diagnostic method for the infection (rapid antigen test, PCR, serology)?
- Why was the diagnostic test performed: due to symptoms, contact with a positive person, screening…?
- Did you have any symptoms during the infection? Was it asymptomatic?
- Which was the first symptom you had during the infection?
- During the infection, did you experience:
    - Fatigue: YES / NO. How many days?
    - Muscle pain: YES / NO. How many days?
    - Fever: YES / NO. How many days?
    - Chest pain: YES / NO. How many days?
    - Cough: YES / NO. How many days?
    - Rhinorrhoea: YES / NO. How many days?
    - Dysgeusia: YES / NO. How many days?
    - Ageusia: YES / NO. How many days?
    - Diarrhoea: YES / NO. How many days?
    - Headache: YES / NO. How many days?
    - Dyspnoea: YES / NO. How many days?
    - Skin rash: YES / NO. How many days?
- Did you need to be admitted to a hospital during the infection?
- If you were admitted to a hospital:
- How many days were you admitted?
- What were the specific treatments given during hospitalization, if any?
    - Ceftriaxone YES / NO
    - Azithromycin YES / NO
    - Kaletra YES / NO
    - Dexamethasone YES / NO
    - Antivirals like Remdesivir YES / NO
    - Hydroxychloroquine YES / NO
    - Plasma YES / NO
    - Interleukin 6 (IL-6) inhibitor YES / NO
    - Anticoagulation: prophylactic dose/full dose YES / NO
- Did you need oxygen therapy?
    - Nasal cannula YES / NO
    - Ventimask YES / NO
    - Oxygen mask with Reservoir YES / NO
    - High-flow nasal cannula (Optiflow) YES / NO
    - Mechanical ventilation/intubation YES / NO
- Did you need ICU admission? How many days?
- During the COVID-19 infection, do you remember having a liver disease decompensation (ascites, edema, hepatic encephalopathy…)?
- After the COVID-19 infection, do you remember having a liver disease decompensation (ascites, edema, hepatic encephalopathy…)? How long after the infection did the decompensation occur?

<u>Figure S1</u>. ROC-curve analysis: Diagnostic performance of SARS-CoV-2 infection for the prediction of decompensation.

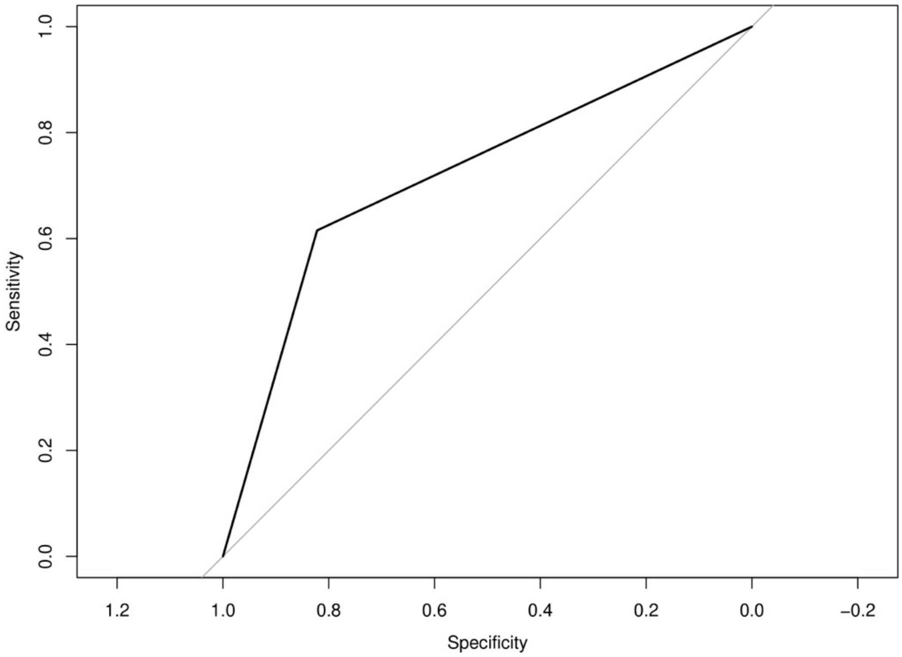

Legend Figure S1: The discriminative ability of the SARS-CoV-2 infection was a moderately good predictor of decompensation (Area under the ROC Curve (AUC) 0.72; 95% CI 0.62–0.82), with a sensitivity of 0.615 (0.406, 0.798) and specificity of 0.822 (0.750, 0.880), to identify patients with decompensation during follow-up.
